# 

**Published:** 1914-01-31

**Authors:** 


### CONTENTS.

PAGE

**The Hospitals and the Coal-Porters' Strike** ... 465

**Rate-Aided Clinical Pathology** ... ... ... 466

**Hospital and Institutional News** ... ... ... 467

The Camberwell Infirmary Libel Case—The New Staff Appointments at Cardiff—Institutional Porters' Fine Records—How a Waiting List May be Reduced—What an Annual Meeting Ought to Be—Penalties for Non-notification—The Public and Chilled Meat—Parents and Operations—The Saturday Fund Awards—The South Shields Guardians and the Use of Alcohol—Damages Against a Nursing Home Proprietor—Co-operation in Public Health Work—A Central Laboratory for London Infirmaries—New Treasurer at Salford Royal Hospital—A Winter in an Ice-cave—Deaths under Anæsthetics—Medical Books at Poor-Law Infirmaries—Resignation of a Hospital Treasurer—The Chelsea and Southwark Dispensary Schemes—A Municipal Dispensary and Guy's Hospital—Leprosy in Australia—The Insurance Act and Pharmaceutical Organisation—This Week's Drug Market—Free Season Tickets for the Scottish Exhibition.

PAGE

**New Developments in Medicine** ... ... ... 473

Modern Methods of Transplantation.

**Research and Remedies** ... ... ... ... 474

**Sir George Newman's Report** ... ... ... 475

The Year's Work in School Medical Inspection.

**A Dental Surgery in a Factory** ... ... ... 476

**St. George's Hospital** ... ... ... ... 477

**The Camberwell Infirmary Case** ... ... ... 478

Dr. W. J. C. Keats Awarded Damages for Libel.

**Gifts and Bequests** ... ... ... ... ... 478

[*See over.*]

### CONTENTS—(*continued*).

PAGE

**Reports on Hospitals of the United Kingdom—Series III.** ... ... ... ... ... ... 479

By Sir HENRY BURDETT, K.C.B., K.C.V.O.

Addenbrooke's Hospital, Cambridge.

**Book Reviews in Brief** ... ... ... ... 480

**The Women's Hospital for Children, Harrow Road** ... ... ... ... ... ... ... ... 481
An Institution Attempting too much.

**Appointments** ... ... ... ... ... 482

**Hospital Economics** ... ... ... ... ... ... 483
The Statistical Report of King Edward's Hospital Fund. IV.

**Hospitals from the Patients' Point of View** ... 484
A Pocketful of Reminiscences.

**The Origin and Evolution of the 18th Century Hospital Movement** ... ... ... ... 485

**Round the World** ... ... ... ... ... ... 486

PAGE

**The Editor's Letter-Box** ... ... ... ... ... 487

Dr. Midelton on Treatment by Continuous Counter-Irritation.

Mr. J. F. Peart, F.R.C.S.I., on the Clinic Movement.

London Staff Appointments.

Article 10 of the Poor-Law Orders.

Patients and their "Beauty Sleep."

Examination Questions for Red Cross Students.

**The Profession of Medicine** ... ... ... 490
The National Medical Union's Latest Undertaking.
More Panel Incidents at Dundee and Wisbech.

**The Institutional Worker** ... ... (Suppl.) 1

Suggestions on Hospital Economy.

**Our Bureau of Information** ... ... „ 2
Employment and Training.
The Sick and in Need.

**Institutional Needs** ... ... ... „ 3

**The Book Market** ... ... .. „ 3

